# Assessing the Effects of Land Use on Surface Water Quality in the Lower uMfolozi Floodplain System, South Africa

**DOI:** 10.3390/ijerph18020561

**Published:** 2021-01-11

**Authors:** Mandla Dlamini, George Chirima, Nebo Jovanovic, Elhadi Adam

**Affiliations:** 1 School of Geography, Archaeology and Environmental Studies, University of the Witwatersrand, Johannesburg 2000, South Africa; Elhadi.Adam@wits.ac.za; 2 Institute for Water Studies, Department of Earth Science, University of the Western Cape, Cape Town 7535, South Africa; njovanovic@uwc.ac.za; 3 Geoinformation Science Division, Agricultural Research Council-Institute for Soil, Climate and Water, Pretoria 0083, South Africa; ChirimaJ@arc.agric.za; 4 Centre for Geoinformation Science, Department of Geography, Geoinformatics and Meteorology, University of Pretoria, Pretoria 0001, South Africa

**Keywords:** crop farming, chemical parameters, soil properties, seasonal variation

## Abstract

This study investigated the impacts of cultivation on water and soil quality in the lower uMfolozi floodplain system in KwaZulu-Natal province, South Africa. We did this by assessing seasonal variations in purposefully selected water and soil properties in these two land-use systems. The observed values were statistically analysed by performing Student’s paired *t*-tests to determine seasonal trends in these variables. Results revealed significant seasonal differences in chloride and sodium concentrations and electrical conductivity (EC) and the sodium adsorption ratio (SAR) with cultivated sites exhibiting higher values. Most of the analyzed chemical parameters were within acceptable limits specified by the South African agricultural-water-quality (SAWQ) water quality guidelines for irrigation except for sodium adsorption ratio (SAR), chloride, sodium and EC. EC, pH and nitrate content which were higher than the specified SAWQ limits in cultivated sites. Quantities of glyphosate, ametryn and imidacloprid could not be measured because they were below detectable limits. The study concludes that most water quality parameters met SAWQ’s standards. These results argue for concerted efforts to systematically monitor water and soil quality characteristics in this environment to enhance sustainability by providing timely information for management purposes.

## 1. Introduction

In southern Africa, wetlands play a significant role in the livelihoods of many people [1,2]. The ability of wetlands to maintain dry season flow provides farmers in semi-arid areas with opportunities to grow crops all-year-round, thereby improving their food security and income. Besides crop production, wetlands provide other services that support people’s livelihoods such as improving water quality, attenuating floods and climate change regulation [3,4]. Wetlands act as sinks for greenhouse gases such as carbon dioxide, which influence local and regional weather patterns [5]. They form a critical component of a region’s water resources in the interface between aquatic and terrestrial environments, as well as between surface and groundwater systems [6]. Wetland ecosystems are extremely valuable not only because they are highly productive but also because they provide habitats for many wild animals and plant species [6,7] and non-material benefits such as spiritual enrichment, recreational and aesthetic services [8].

Despite the provision of the above valuable services and functions, wetlands are globally among the most threatened ecosystems. As in many parts of the world, the extent of South African wetlands continues through conversion to cropland [9]. They are also adversely affected by the impacts of water extraction, urbanization, infrastructure development, pollution, poor farming practices, and climate change and variability [8,10,11,12,13,14,15]. In the arid and semi-arid areas, for example, wetlands have been severely degraded through conversion to croplands. This degradation has been aggravated by the persistent loss of ecosystem services, impairment of different functional processes, water quality, and loss of biodiversity. The trade-off between crop production and water quality is one of the most serious and challenging issues in modern agricultural landscapes [16].

One of the main problems associated with wetland water quality management in South Africa is eutrophication due to nutrient enrichment by agricultural activities [17] the most important of which include agrochemicals that are widely used to increase crop production. However, the intensive application of agrochemicals can pollute water bodies through nutrient enrichment via surface runoff, leaching and sub-surface flow [18,19,20]. Fertilizers and a wide range of different agrochemicals affect the physico-chemical characteristics of soil, surface water and biota [19]. Nitrate pollution, for example, is common in agricultural regions throughout the world where eutrophication is increasingly becoming one of the major environmental problems [21] with water from farmed areas often exhibiting high nutrient loads [22]. In these agricultural regions, crop production activities impose severe environmental stresses that affect functional processes in wetland ecosystems by (a) polluting potable water resources, (b) disrupting, natural nutrient cycling processes, soil retention and carbon sequestration and (c) reducing biodiversity. In addition, environmental degradation through agricultural related deforestation can also affect vegetation with irrigation infrastructure aggravating the situation by modifying surface and in-stream flows, infiltration patterns and overall water quantity.

Floodplain wetlands in many parts of the world have been used for agriculture because of their high fertility, which is sustained by flood-driven deposition of natural nutrients. This natural endowment renders wetland ecosystems susceptible degradation by attracting a wide-ranging of resource extractive practices [23]. In South Africa, most wetlands are confronted by severe threats because of their suitability for agriculture and the numerous ecosystems goods and services they provide [24]. These scenarios are also evident in other African countries with Nigeria, Rwanda and Ethiopia, for example, going as far as encouraging farmers to cultivate wetlands to mediate drought-induced food shortages [25]. Although several studies in these countries have reported the importance of wetlands in supporting the production of rice, maize, and various vegetable crops for both subsistence and commercial use [25,26,27,28] their sustainability is being compromised by inappropriate human resource use practices.

The same threats are becoming increasingly evident in the uMfolozi floodplain/wetland system, which is situated in the northern peripheries of South Africa’s KwaZulu-Natal province. This wetland resides in iSimangaliso Wetland Park, which is well known for being, a World Heritage site that has a scenic environmental layout, which supports local communities by attracting wide-ranging tourism activities. The same wetland also supports its inhabitants by sustaining commercial and subsistence crop farming activities. Having recognized the vital importance of this wetland to the livelihoods of local communities in this environment, we decided to assess water quality in this locality to ascertain how human interference is impacting on this floodplain ecosystem. This motivation was inspired by a conscious acknowledgement of the fact that comprehensive wetland water quality monitoring is essential because it provides the vital information needed in formulating sound management practices for this threatened wetland ecosystem. In South Africa, water quality guidelines stipulate the target water quality range (TWQR) for different water use sectors such as agriculture [29]. The TWQR can be defined as the concentration levels at which certain constituents would cause adverse effects on the fitness of water for different consumptive and non-consumptive uses in a manner that does not compromise sustainability [30].

To the best of our knowledge, there is a paucity of long-term water quality datasets on wetlands in South Africa, although there is an existing monitoring protocol. Such data helps to elucidate what constitutes good and bad water quality concerning anthropogenic activities and the natural environment. Therefore, providing valuable knowledge that is important to inform the formulation of implementable management interventions. This realization prompted us to evaluate the quality of the lower uMfolozi floodplain system based on systematic characterization and assessment of the impacts of surface water discharged from irrigated agriculture on the wetland system’s water quality. This evaluation was guided by the hypothesis that cultivated areas have higher concentrations of physico-chemical parameters such as nitrates, electrical conductivity (EC), sodium adsorption ratio (SAR) and other parameters, compared to uncultivated sites. The objectives were to, (i) to assess the physico-chemical characteristics of water in the lower uMfolozi floodplain wetlands based on South African water quality guidelines for agricultural water use in irrigation, and (ii) to evaluate the seasonal impacts of irrigation agriculture on water quality.

## 2. Materials and Methods

### 2.1. Study Area Background

**Figure 1:**
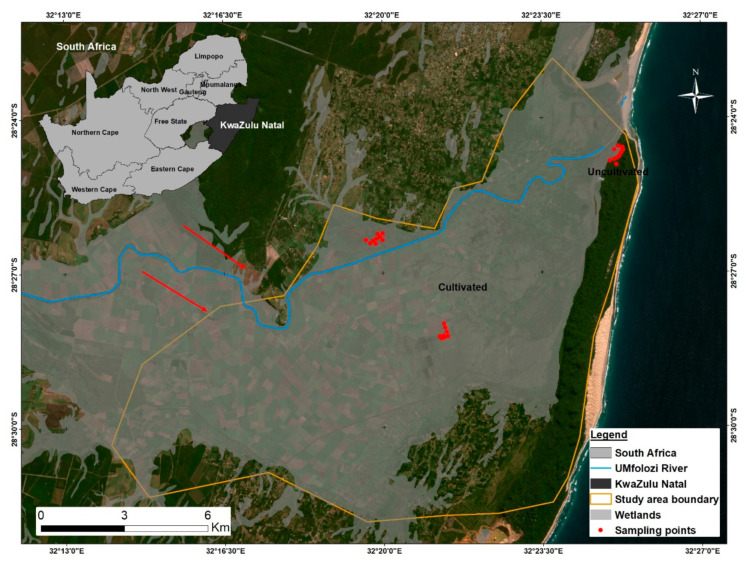
Study area: The uMfolozi floodplain in uMkhanyakude District Municipality. Red arrows show west to east water flow towards the Indian ocean.

The uMfolozi floodplain is situated in the vicinity of St Lucia, a town located in uMkhanyakude District Municipality (28°22′ S and 32°25′ E), KwaZulu-Natal Province, South Africa (Figure 1).

This municipality is one of the poorest and most underdeveloped local authorities in South Africa [31]. The uMfolozi floodplain is approximately 19,000 hectares in extent [32]. Physiographically, the floodplain is bound inland by the incised meanders of the uMfolozi River. The river cuts through rhyolite rock of the Lebombo to the east by the Indian Ocean, to the south and north by Zululand and Maputaland formation rocks [33]. Maputaland, which covers most of the study area, comprises calcarenite, clayey sand, limestone and conglomerate. The region experiences a subtropical climate, with large seasonal variations in precipitation ranging between 671 and 1002 mm [34]. Rainfall in the region of the coastal uMfolozi floodplain averages 1090 mm per annum, declining to 645 mm pa towards the upper catchment boundary. The region experiences summer rainfall, with January and March being the wettest months [34]. Table S1 shows the monthly average rainfall and maximum temperatures for the 2017 and 2018 periods. Therefore, the inundation of the floodplain is seasonal. Potential evapotranspiration on the floodplain is 1805 mm pa [35].

The surface hydrology is comprised of two major tributaries, the Black uMfolozi which arises in the north approximately 1500 m a.m.s.l and the more southerly White uMfolozi which arises at an altitude of 1620 m. The two rivers join approximately 50 km west of the uMfolozi River’s mouth to the sea. The dominant vegetation types in the floodplain system include *Cyperus papyrus*, *Phragmites mauritianus*, *Phragmites australis* and *Ficus trichopoda*. The uMfolozi River has a highly variable flow regime [36], characterized by a low base-flow and a few large but brief floods each year. Floods are a feature of the river and they have a profound influence on the geomorphology of the floodplain [37]. Although infrequent and of short duration, these floods carry much of the annual river flow and it is during these times that most of the geomorphologic changes occur.

The major source of livelihood for the majority of people in this area is agriculture with sugarcane being the primary crop produced by most farmers. The clearance of land for cultivation of sugarcane and commercial forestry dates back to 1911 [38]. By 1960, more than 50% of the floodplain had been converted to sugarcane farms whose establishment was made possible by the construction of canals, diversion of the uMfolozi River and the clearing of indigenous wetland vegetation [31,36]. The floodplain also supports subsistence farming of other crops wherein the dominant ones include sweet potato (*Ipomoea batatas)*, banana (*Musa acuminate)*, and amadumbe (*Colocasia esculenta*). Land conversion takes place through direct clearance of vegetation by using the traditional slash and burn techniques, which date back to 2006. In 2009 the herbicide (imidacloprid) was first introduced in the study area [39] to suppress the pest called sugarcane Thrip (*Fulmekiola serrata*) which was identified in 2005 [40]. In terms of fertilizer applications, sugarcane requires substantial quantities of nitrogen (N) and potassium (K) for optimum yields with N being applied through direct injection into the soil to depths of ~200 mm. These agricultural activities make the floodplain susceptible to the pollution which compromises the livelihoods of local communities by adversely impacting on the ecological integrity and health of the entire system.

### 2.2. Data Collection

Three study sites (two with cultivation and one pristine uncultivated site) were selected in the floodplain wetland area (Figure 1). The two cultivated sites were approximately 3 km apart and are used for commercial and subsistence crop farming. The commercial site comprised sugarcane while the subsistence site consisted of sweet potato, amadumbe and banana. The sweet potato, amadumbe and banana are grown on land that is temporarily flooded. Subsistence crops are not irrigated while sugarcane is supplemented with sprinkler irrigation using water from the uMfolozi River. The uncultivated site (control site) was situated in Maphelane Nature Reserve (where there is controlled access), approximately 8 km away from the cultivated plots and closer to the ocean. Sampling sites were selected based on land use as well as on accessibility, safety, and the presence of surface water.

Four site visits were undertaken during 2017 and 2018. Surface water samples were selected and collected randomly based on the anthropogenic activity. Water samples were taken between 8 a.m. and 1 p.m. [41] at a distance of ±100 m intervals. Water depth was 0.85 m during the wet season and ± 0.50 m on a dry season. The number of samples collected depended on the presence of surface water during the wet (March) and dry (July) periods. Therefore, the number of samples ranged between 13 and 17 from the cultivated sites and 10 from the uncultivated site. Water samples were collected using 100 mℓ acid pre-treated sampling bottles at every sampling point [42]. Temperature, pH and electrical conductivity (EC) were measured on-site using a handheld multi-parameter, YSI professional plus [42]. Accuracies for each probe were according to YSI Incorporated, 2011 standards (conductivity: 0.5% or 0.001 mS/cm, pH: ±0.2 and temperature: ±0.2 °C). Global positioning system (GPS) coordinates of each sampling point were recorded so that sampling could take place at the same points during subsequent field trips.

Soil samples were collected at the same locations as water samples in March and July of 2017 and 2018. The samples were collected to a depth of 50 cm at intervals of 0–10, 10–20, 20–30, 30–40 and 40–50 cm following [43] using a soil auger. Soil samples were mixed at each depth to form one composite sample. They were then transported to the laboratories and air-dried at room temperature for the chemical analyses. Once-off soil samples (July 2018) were collected from the same locations mentioned above for herbicide analysis.

### 2.3. Water and Soil Lab Analyses

The water and soil properties were determined in the laboratory (chemical analyses) and used to assess the extent of environmental degradation due to agriculture via comparing cultivated and uncultivated sites. The concentrations of cations were determined by the ion chromatography method with a conductivity detector (Shimadzu, Tokyo, Japan). The environmental protection agency (EPA) method was used [44] to determine anion concentrations. A volume of 2–3 mL was injected into an ion chromatograph where ions of interest were separated and measured using a guard and the analytical column. Both anions and cations were determined using an ICPE-9000 spectrometer. Anions detection limits were as follows: boron (0.01), fluoride (0.030), nitrite (0.022), nitrate (0.060) and phosphate (0.109).

Soil pH was measured in a 1:2.5 soil/water ratio using a glass rod pH meter calibrated using buffer solutions of pH 4, 7 & 8. Cation exchange capacity (CEC) was determined following the extraction method where soil samples were also saturated with ammonium acetate buffered at pH 7 to displace the exchangeable bases [45]. To determine N-NO_3-_, 1M HCl was used to extract nitrate from the soil mass to volume ratio of 1:10 and determined with a flow analyzer. The soil was saturated with deionized water for 8 h and the filtrate analyzed for alkalinity and EC. To analyze the organic matter, a known sample mass was dried at 100 °C for 8 h to calculate the moisture percentage. The sample was then ashed at 6000 °C and the organic matter percentage was calculated by employing loss on ignition (LOI). A CNS-2000 analyzer (Leco Corporation, St Joseph, MI, USA) was used for total carbon+nitrogen determination in soil samples, based on the dry combustion principle. Soil samples were analyzed at combustion temperatures of 950 °C [46]. The Bouyoucos-hydrometer method explained by [47] was used for soil texture determination.

Glyphosate, imidachloprid and ametryn were quantified using standard lab methods [48] with a liquid chromatography-mass spectrometer (LC-MS) while ametryn was quantified using a gas chromatography-mass spectrometer (GC-MS). The limit of quantification (LOQ) for imidachloprid and ametryn was 0.01 mg/kg, and 0.05 mg/kg for glyphosate.

### 2.4. Statistical Analysis

Data were compared between cultivated and uncultivated sites to evaluate the impact of agriculture on soil and water quality. In addition, data collected in different seasons (dry and wet) were compared to determine seasonal variability of water and soil quality. Paired *t*-tests were performed in IBM SPSS Statistics version 26 (SPSS Inc., Chicago, IL, USA) in order to determine if differences in water and soil parameters were significant. The observed water parameters were compared to the South African water quality guidelines for agricultural water use in irrigation [49] to determine the fitness of the wetland water for use. For this purpose, field recorded water parameters were compared to the accepted limits for each parameter (Table S2). The scientific information underpinning the specification of pollution limits can be found in [49] Seasonal data for EC and sodium adsorption ratios (SAR) by [29] were ranked against the irrigation water classes for EC and SAR provided in Table S3. Hypothesis testing approaches (*t*-tests) were performed to determine significant deviations of parameters measured from the field to the South African water quality (SAWQ) guidelines for irrigation.

## 3. Results

### 3.1. Comparison of Water Quality Analyses between Cultivated and Uncultivated Sites and Seasonal Dynamics

The results of water quality analyses for cultivated and uncultivated are presented in the form of a table (Table 1).

As shown in Table 1, chloride showed higher concentrations in cultivated sites (*p,* 0.020) compared to uncultivated sites. A similar pattern was observed for sodium adsorption ratio in both wet and dry periods (*p*, 0.014 and 0.009) and sodium (*p*, 0.017 and 0.003), exhibiting higher values in the cultivated compared to the uncultivated sites. Significant differences were also observed in the wet period for pH (*p*, 0.038) and electrical conductivity (EC) with higher concentrations from the cultivated site. The rest of the parameters when detected such as ammonia, phosphate, nitrate and boron did not show any significant differences between cultivated and uncultivated sites.

A comparison of water quality parameters between the two seasons (wet and dry) was explored. However, due to equipment failure in the field, analysis for pH, EC for March 2018 was done in the laboratory with no results for the temperature of the period. Water recordings indicated a clear seasonal change with an average temperature of 26.45 °C (higher) recorded in the wet summer season and 19.84 °C in the dry winter season. *T*-tests showed that the concentrations of water quality from the sampling seasons varied among the parameters. Ammonium showed significant differences between the seasons in both cultivated and uncultivated (*p*, 0.006) sites. In both cultivated and uncultivated sites, chloride (*p* ≤ 0.05), sodium adsorption ratio (*p* ≤ 0.05) and sodium also showed significant differences between the seasons. The significant difference (*p*, 0.011) for EC and pH (*p*, 0.048) between the seasons was observed only in the uncultivated sites.

### 3.2. Comparison of Water Quality with South African Water Quality (SAWQ) Guidelines for Irrigation

**Figure 2:**
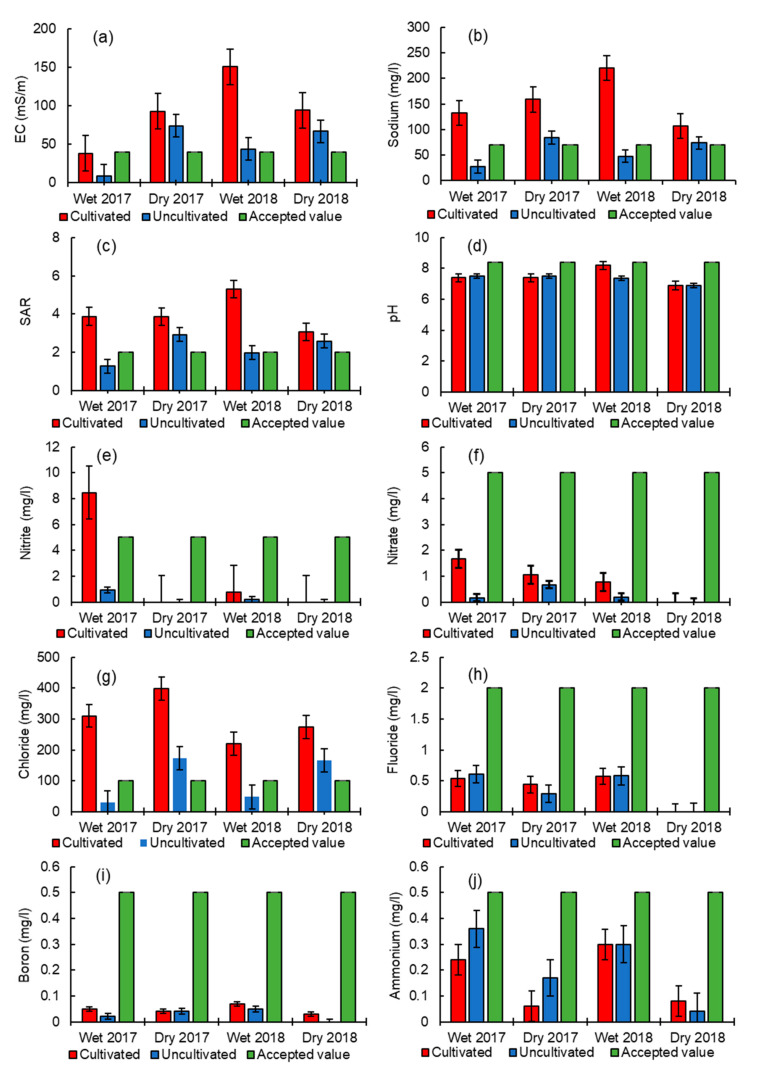
Comparison of water quality constituents recorded in wetlands (uncultivated and cultivated) with South African water quality (SAWQ) target values in wet and dry seasons of 2017 and 2018, (**a**) EC, (**b**) Na, (**c**) SAR, (**d**) pH, (**e**) NO_2_^−^, (**f**) NO_3_^−^, (**g**) Cl^−^, (**h**) F, (**i**) B and (**j**) NH₄⁺.

Figure 2 shows results from comparative analysis of field derived samples and SAWQ guidelines for irrigation. The results correspond to the mean of the parameters using the error bars from the standard deviation values. Overlaps of the error bars implied the insignificant differences in parameters between cultivated and uncultivated.

As shown in Figure 2, the seasonal means of each parameter were compared against SAWQ target concentration levels for agricultural use (irrigation). It should be noted that not all the parameters analyzed in this study are found in the SAWQ guidelines and as explained earlier in Section 3.1, EC and pH in the wet season of 2018 were measured in the laboratory because the instrument failed during field investigation. Most of the parameters were within the acceptable levels in both the cultivated or uncultivated sites, except for EC, SAR, sodium and chloride. It was observed with 95% confidence that the actual means of ammonium, boron, fluoride, pH, nitrate and nitrite from the fieldwork did not differ significantly from the values in the SAWQ guidelines (Table 1). Although nitrite concentrations were within the acceptable limits, a sharp spike of 8.7 mg/L was observed in the wet period in the cultivated site for March 2017, which exceeded the SAWQ guidelines. Nitrite is highly mobile [50], therefore its high loading from the increased agricultural activities and high rainfall that occurs during wet seasons could be an important factor. As observed from the results, spikes of this nature were not seen for the dry season of the year 2017 and the rest of the study periods.

EC exceeded the target concentrations in July of 2017 and March of 2018 for both uncultivated and cultivated sites. For the periods that exceeded the limits, cultivated samples were more than 50% higher while the uncultivated samples were approximately 40% higher than the SAWQ targets. SAR exceeded the target concentrations for all periods in the cultivated sites, while the exceedance only occurred in the dry season (July) of 2017 and 2018 from the uncultivated site. SAR samples for the cultivated sites were more than 50% higher with less than 50% from the uncultivated site compared to the irrigation targets. Sodium and chloride exceeded the SAWQ guidelines limits in cultivated sites for all the study periods. A similar pattern as of SAR for sodium and chloride from the uncultivated site was observed where exceedance only occurred in the dry periods. Sodium samples from the cultivated sites were two to three times higher and less than 10% higher from the uncultivated site compared to SAWQ guidelines limits. The chloride content in samples from the cultivated sites was three to four times higher whereas in the uncultivated sites they were almost two times higher than the SAWQ limits.

### 3.3. Cultivation and Soil Quality

Clay and sand content of the soil samples analyzed using an independent two-sample *t*-test from uncultivated and cultivated sites were significantly different (*p* ≤ 0.05), while silt content was not significantly different (Table 2). The cultivated site had a higher mean value of clay (44.60) than an uncultivated site (9.35). On the contrary, the cultivated site had a lower mean value of sand (13.43) than an uncultivated site (73.63). Considering the location of the uncultivated site in the vicinity of sand dune depositions (Figure 1), the sand content was expected to be high and the clay content to be low.

In terms of the herbicides analysis, no detection of glyphosate, imidacloprid, ametryn in the soil samples was observed as all the herbicides were below the limit of quantification mentioned in Section 2.3.

Table 3 shows that organic matter, cation exchange capacity, total carbon and nitrogen had higher values in the uncultivated site but there were no significant differences in all periods. Both pH and EC exhibited higher values in the cultivated sites but again there were no significant differences. Nitrate content was higher in the cultivated sites and differed significantly between the sites

## 4. Discussion

This study involved assessments of water quality to ascertain the impacts of surface water discharged from irrigated agriculture on wetlands water quality. Furthermore, the concentrations of chemical parameters were compared to agricultural guidelines of South Africa for fitness for use. Additionally, the study evaluated the impacts of cultivation on water quality of the floodplain wetland during dry and wet periods. Parameters on the cultivated sites exhibited higher concentrations of salts throughout the study period compared to the uncultivated sites. These include chlorides, sodium, sodium adsorption ratio and EC. These findings are in agreement with observations by [51] who report higher nutrient loads in farmed areas in The Netherlands. This appears to be a regional and global concern over wetlands and water quality. Most of the chemical parameters assessed were within permissible limits for water quality for agricultural use, except for electrical conductivity (EC), sodium adsorption ratio (SAR), sodium and chloride. This has important implications for irrigators as it may cause accumulation of salts (mainly sodium and chloride) and salinization of soils and water in the long run. In our study, a significant relationship existed between the floodplain chemistry and season. For instance, ammonia, SAR, chloride and sodium showed significant differences within the seasons both in cultivated and uncultivated sites. This suggests that seasonal variations in both anthropogenic and natural processes such as precipitation influence the quality of wetlands water to different attributes between seasons.

A high EC can stress plants and cause productivity losses. In the present study, water conductivity ranged from 9 to 150 mS/m between the years 2017 and 2018 (Table 1). Obtained values were lower in the site with no cultivation as compared to the cultivated sites. An EC of 40 mS/m is accepted by SAWQ guidelines as a threshold concentration level for agriculture (irrigation). In this study, all the EC values from the cultivated marginally exceeded the guideline, while from the uncultivated site only values that fell under the dry season were above the limit (Figure 2). High salt concentrations in the water or soil can reduce the portability of water and adversely affect plant productivity [52]. Furthermore, high EC can cause the inability of vegetation to compete for ions in water and soil causing toxicity of particular ions [53] such as chloride, sodium, etc. However, in terms of irrigation classes for fitness use [51], EC fell between good and marginal values. Irrigated agriculture through water abstraction can reduce the dilution capacity as well as potentially add significant quantities of salt via return flows and the concentrating effect of increasing evapotranspiration. A significant difference between cultivated and uncultivated sites in EC in the wet season was found in the study.

This can be attributed to the surface runoff thereby dissolved ions by rain and floodwater. A different trend was reported in a study in India where EC was found to increase during the monsoon [54]. Shabalala, et al. [55] confirmed EC to be higher in the wet season than in the dry season in Bonsma Dam located in the southern Drakensberg, KwaZulu-Natal. This was attributed to the water bodies being recharged during the rainy season, with salts and nutrients being leached to the water table. On the other hand, Mmualefe and Torto [56] reported dilution in the wet season. Furthermore, the increase in EC can be caused by factors such as geology and soils of the area [55] as well as land use like agriculture. Elsewhere in Uganda, the increase in EC in a dry season was caused by the evaporation of water from the underground water channels which increased the concentrations of dissolved salts [57]. High concentrations of chloride which was the case in this study area are typically associated with the magnitude and extent of manure use and soil infiltration [58] are known to increase electrical conductivity [59,60]. Therefore, the observed increase in EC from cultivated sites is a result of agricultural inputs and high evaporation, which leads to the concentration of salts.

The geology of the study area comprises calcarenite, clayey sand, limestone, conglomerate, and dune sand. Clay soils tend to have high EC as a result of the presence of salts that dissolve when washed into the water and are retained in the soils with inherently low hydraulic conductivity [61]. This may explain the high conductivity of cultivated floodplain wetlands. Although this study did not investigate the origin of the salt in the study area, the literature confirms that EC is primarily affected by the geological soil and rock formations of the area through which water flows [62,63]. For example, freshwater ecosystems that run through areas with granite bedrock tend to have a lower EC as its composition has more inert materials that do not ionise [61]. The tidal forcing effect is also a common feature in coastal environments, enhancing the extent of saltwater ingress particularly near the top of the water table [64]. The mean concentrations of chloride were above the SAWQ limits in the cultivated sites and above the same limits in the uncultivated areas during the dry period. The possible source of chloride in this study could be attributed to the runoff water from agricultural fields and irrigation in which can increase chloride concentrations. This finding is consistent with observations by [65,66] who report an increase in chloride concentrations from fertilizer applications in agricultural. The pH of most freshwater systems is predominantly determined by or dependent on atmospheric influences, the mineral content of the surrounding rocks, soils and other landforms. It often ranges from 6 to 8, which is ideal for aquatic life [67,68]. pH values ranging between 6.5 and 8.4 are considered acceptable by the SAWQ guidelines for agricultural use. A significant difference in pH concentrations between cultivated and uncultivated areas can be attributed to land clearing activities that induce salinity problems. In this study, water was alkaline (pH > 9). A high pH (7.44) in the wet season compared to 7.20 in the dry season (Table 1) could be attributed to the surface runoff of agricultural input. On the contrary, Brainwood, et al. [69] observed a different pattern of an increase in pH associated with a decrease in rainfall and an increase in water use for irrigation. The pH of water bodies can be affected by factors such as bedrock and soil composition through which water moves. Some rock types such as limestone can neutralise acidity, while others like granite do not affect the pH [70]. Most natural waters have a pH suitable for irrigation because it tends to be buffered by the soil under pristine conditions and most crops can tolerate a reasonable pH range [71].

Nutrients, mainly nitrogen and phosphorus, are applied to croplands in the form of fertilizers to promote crop growth. An excessive amount of these nutrients can cause water quality problems when entering water environments. In the current study, most ionic concentrations such as ammonium, boron, fluoride, nitrate, etc. were within the acceptable water quality range for agricultural use (irrigation) with the exception of sodium and chloride. Lower values of ammonium from the cultivated sites were attributed to the adsorption of clay minerals and therefore less mobile, while high nitrate in the cultivated sites was attributed to its high mobility. SAR determines the suitability of water for irrigation purposes because it is linked to the sodium hazard in soils [72]. The set limit for SAR by SAWQ guidelines is two. In this study, most of the SAR values exceeded the set limit (Figure 2); they fell between good and marginal classes (Table 2), indicating water of medium to high salinity. This means water infiltration problems that are caused by the swelling of clay minerals which weakens the soil aggregates by closing soil pores [73] may occur in this area in future. High sodium values with a corresponding increased level of SAR may be caused by the precipitation of magnesium and calcium as carbonates and bicarbonates leaving sodium in solution [74]. Rahman, et al. [75] reported high concentrations of calcium, chloride and magnesium due to seawater intrusion in Sundarbans coastal wetlands in Bangladesh. Our study area is situated in a similar environment along the coast and close to the ocean where nutrient inputs into coastal water may occur naturally through processes such as oceanic upwelling [76]

Physico-chemical soil analyses including texture, organic matter, total carbon and nitrogen, cation exchange capacity (CEC) and nitrate were assessed in Table 3. Changes in soil properties through tillage can affect soil and water quality simultaneously. Nutrients mostly found in the topsoil can be transported through surface runoff, reaching water bodies such as wetlands. These nutrients are manifested in the degradation of surface water quality. Variations in the physico-chemical properties of soil and water between cultivated and uncultivated sites were observed in our study area. The results for the CEC showed higher values in the uncultivated sites, albeit not significantly different between the sites. Average CEC values for uncultivated sites ranged between 25.9 cmol (+)/kg and 37.8 cmol (+)/kg while in the cultivated sites they were between 19.8 cmol (+)/kg and 25.9 cmol (+)/kg. These findings concur with [77,78] who found CEC to be higher in the undisturbed soils compared to the cultivated ones. The high CEC in the uncultivated sites could be explained by high organic matter (11.5% and 11.7%) compared to the cultivated soil (8.5% and 8.7%) and related to land cultivation decreasing organic matter. Leaching and low vegetation of cultivated wetlands that expose the soil to direct contact with surface runoff is another reason for lower CEC content in cultivated sites [79,80]. The pH was higher in the cultivated sites but differences were not significant. As indicated by [81], the removal of vegetation and exposure of soils to wind and sun could increase aeration and temperature of the soil. This could facilitate the rapid decomposition of organic matter and prevent the accumulation of acids, thereby increasing pH [82,83]. Lower pH values in the uncultivated soil could be attributed to the release of organic acids from organic matter decomposition. Concentrations of total nitrogen in the uncultivated sites differed from the cultivated ones, although not significantly. These differences were expected because of the high organic matter in the uncultivated sites which is the main source of nitrogen [84]. A decrease in vegetation cover, soil preparation and wetland drainage can cause a decrease in the total nitrogen content. Previous studies have also shown similar results with cultivation of wetland being associated with increased soil aeration and temperatures that accelerate rapid decomposition and mineralization of plant materials thereby lowering the nitrogen content [83,85]. Crops in the surface soil can absorb and retain pollutants from cultivated land, thereby influencing water quality. Sodium, chloride, EC and SAR from water analyses were higher in the cultivated land and exceeded the SAWQ guideline limits. These results corresponded with soil analysis findings, which showed lower organic matter, CEC, total nitrogen and carbon in the cultivated sites with high EC and pH. The clearing, fertilization and tillage practices for cultivation purposes can increase nutrient concentrations in the soil. These nutrients are usually controlled and reduced by natural vegetation and grasslands, thereby regulating water quality.

## 5. Conclusions

In light of the set objectives and hypothesis, some important conclusions can be drawn. This study hypothesized that cultivated sites would exhibit higher concentrations of chemical parameters comprising nitrate, nitrite, boron, ammonia, pH and electrical conductivity (EC) compared to sites without cultivation. However, only a few water quality parameters like chloride, sodium adsorption ratio (SAR), nitrate and sodium showed significant differences in terms of land use (cultivated and uncultivated) and seasonality. Most water quality parameters were within acceptable limits of fitness for agricultural use, except EC and SAR, which was attributed to anthropogenic inputs, seasonality and geological weathering. Regardless of their exceedance above guideline limits, water EC and SAR fell between good and marginal irrigation classes, suggesting moderate suitability for irrigation. Long-term issues of salinity and sodicity presented by SAR and EC may be expected and this needs to be monitored. These findings have demonstrated that the use of floodplain wetlands for commercial and subsistence crop farming can have minimal impacts on water quality as long as the degree of intensification, pesticide/herbicide and fertilizer use remains within limits.

The conditions of a water body fluctuate periodically. It is therefore recommended that future work should include sufficient surface and groundwater data by employing regular sampling and analysis. The quality and fitness of water vary in tandem with changes in land use, rainfall and farming activities. Therefore, their overall state should be investigated and evaluated. This will aid in understanding surface and groundwater interaction to obtain a complete water resource assessment in the lower uMfolozi floodplain system. It will also be good to compare the study results with the current uMfolozi resource water quality targets other than with South African water quality guidelines for agriculture. However, there was no water quality data available for this catchment. Lastly, although the water quality in the lower uMfolozi is still in a good state, there is a possibility of deterioration in the future as a result of the increasing crop farming activities. A wetland monitoring programme for water quality by agronomists and ecologists is recommended. A monitoring protocol has been available for some years but there is a lack of systematic observation. Deploying an effective monitoring mechanism is therefore recommended in order to provide reliable information that can be used to guide policy formulation and appropriately informed management strategies. This will go a long way in securing the sustainability of this wetland for benefit of present and future generations.

## Figures and Tables

**Table 1:** Water quality parameters based on the land use (cultivated and uncultivated) for 2017 and 2018 dry and wet periods.

Variables	Statistics	WET SEASON CULTIVATED			WET SEASON UNCULTIVATED			DRY SEASON CULTIVATED			DRY SEASON UNCULTIVATED		
		* Mar 17–** Mar 18 Cult			* Mar 17–**Mar 18 Uncult			* Jul 17–** Jul 18 Cult			* Jul 17–** Jul 18 Uncult		
		* N = 17	** N = 17	Avg	* N = 10	** N = 10	Avg	* N = 14	** N = 13	Avg	* N = 10	** N = 10	Avg
NH_4_^+^ (mg−/L)	Mean	0.24	0.31	0.28	0.36	0.34	0.33	0.06	0.08	0.07	0.17	0.04	0.105
	Maximum	0.93	0.79	0.86	0.70	1.66	1.2	0.30	0.27	0.29	0.96	0.12	0.56
	Minimum	0.08	0.08	0.08	0.15	0.05	0.1	0.01	0.01	0.01	0.01	0.02	0.015
	Median	0.15	0.27	0.225	0.34	0.07	0.21	0.05	0.04	0.05	0.08	0.02	0.05
	SD	0.21	0.21	0.21	0.17	0.57	0.38	0.07	0.09	0.08	0.30	0.04	0.17
	*p*-value *	0.163						0.186					
	*p*-value **	0.006						0.006					
B(mg/L)	Mean	0.05	0.07	0.06	0.02	0.06	0.04	0.04	< 0.01	0.025	0.04	< 0.01	0.025
	Maximum	0.15	0.10	0.125	0.04	0.07	0.06	0.05	< 0.01	0.03	0.06	< 0.01	0.035
	Minimum	0.03	0.05	0.04	0.00	0.03	0.02	0.02	< 0.01	0.015	0.02	< 0.01	0.015
	Median	0.04	0.06	0.05	0.02	0.06	0.04	0.04	< 0.01	0.025	0.04	< 0.01	0.201
	SD	0.03	0.01	0.02	0.02	0.01	0.02	0.01	< 0.01	0.01	0.01	< 0.01	0.01
	*p*-value *	0.108						0.359			*-*		
	*p*-value **	0.807						0.000			*-*		
Cl^−^ (mg/L)	Mean	310.36	435.36	265.28	30.07	67.30	38.99	397.71	273.88	335.8	172.87	166.61	169.74
	Maximum	417.18	857.59	373.1	50.98	84.50	52.90	622.49	303.30	462.9	221.82	183.78	202.8
	Minimum	26.4	337.43	109.2	20.27	40.14	27.75	188.91	159.12	174	123.70	123.20	123.45
	Median	349.5	377.26	275.75	23.9	71.13	36.85	379.85	284.05	332	179.68	170.66	175.2
	SD	107.70	134.55	121,13	12.63	16.66	14.65	153.83	41.24	97.5	32.73	18.26	25.495
	*p*-value *	0.020						0.020					
	*p*-value **	0.004						0.012					
NO_3_^−^ (mg/L)	Mean	1.57	0.83	1.19	0.18	0.20	0.19	1.05	< 0.060	0.555	0.71	< 0.060	0.385
	Maximum	5.96	3.27	4.49	0.36	0.50	0.43	3.44	< 0.060	1.75	2.88	< 0.060	1.471
	Minimum	0.05	0	0.03	0.06	0.06	0.06	0.10	< 0.060	0.08	0.14	< 0.060	0.1
	Median	0.67	0.65	0.60	0.16	0.09	0.15	0.57	< 0.060	0.315	0.37	< 0.060	0.215
	SD	1.88	0.78	1.33	0.10	0.18	0.14	1.12	< 0.060	0.59	0.88	< 0.060	0.47
	*p*-value *	0.135						0.056			*-*		
	*p*-value **	0.798						0.001			*-*		
NO_2_- (mg/L)	Mean	8.69	< 0.022	4.46	1.05	0.23	2.76	< 0.022	< 0.022	0.022	< 0.022	< 0.022	0.022
	Maximum	24.10	< 0.022	12.16	2.77	0.93	1.85	< 0.022	< 0.022	0.022	< 0.022	< 0.022	0.022
	Minimum	0	< 0.022	0.111	0	0.00	0.00	< 0.022	< 0.022	0.022	< 0.022	< 0.022	0.022
	Median	9.90	< 0.022	5.061	0.85	0.00	0.43	< 0.022	< 0.022	0.022	< 0.022	< 0.022	0.022
	SD	7.60	< 0.022	3.91	1.16	0.35	0.76	< 0.022	< 0.022	0.022	< 0.022	< 0.022	0.022
	*p*-value *	0.085						0.000					
	*p*-value **	0.001						0.187					
PO_4_^3−^(mg/L)	Mean	0.93	1.46	1.17	0.15	0.49	0.33	2.05	< 0.109	1.08	1.90	< 0.109	1.00
	Maximum	8.01	4.25	6.15	0.24	0.87	0.57	4.00	< 0.109	2.06	3.65	< 0.109	1.88
	Minimum	0.00	0.00	0.00	0.05	0.18	0.13	0.86	< 0.109	0.49	0.43	< 0.109	0.27
	Median	0.12	1.26	0.58	0.17	0.46	0.34	1.71	< 0.109	0.91	2.12	< 0.109	1.11
	SD	2.05	1.44	1.75	0.07	0.24	0.16	0.85	< 0.109	0.48	0.99	< 0.109	0.55
	*p*-value *	0.189						0.621			*-*		
	*p*-value **	0.002						0.000					
F (mg/L)	Mean	0.53	0.56	0.55	0.64	0.58	0.61	0.44	< 0.030	0.24	0.28	< 0.030	0.16
	Maximum	1.24	1.22	1.21	0.80	0.76	0.78	1.26	< 0.030	0.65	0.41	< 0.030	0.22
	Minimum	0.21	0.27	0.24	0.43	0.41	0.41	0.15	< 0.030	0.09	0.13	< 0.030	0.08
	Median	0.50	0.51	0.51	0.63	0.58	0.59	0.35	< 0.030	0.19	0.31	< 0.030	0.17
	SD	0.29	0.26	0.28	0.12	0.13	0.13	0.28	< 0.030	0.16	0.10	< 0.030	0.07
	*p*-value *	0.629						0.180			*-*		
	*p*-value **	0.065						0.000					
pH	Mean	6.69	8.19	7.44	6.64	7.36	7	8.11	7.93	7.15	7.74	7.74	7.20
	Maximum	6.89	9.20	8.01	6.86	7.85	7.33	8.58	8.39	7.75	8.60	7.92	7.80
	Minimum	6.46	7.33	6.90	6.43	7.13	6.77	7.59	7.59	6.55	7.25	7.58	6.80
	Median	6.44	7.94	7.19	6.63	7.28	6.96	7.94	7.94	7.25	7.64	7.73	7.20
	SD	0.15	0.53	0.34	0.19	0.22	0.21	0.27	0.22	0.25	0.46	0.13	0.30
	*p*-value *	0.038						0.305					
	*p*-value **	0.100						0.048					
SAR	Mean	3.71	5.24	4.51	1.26	1.97	1.62	3.86	3.07	3.47	2.88	2.58	2.73
	Maximum	4.77	6.96	5.85	1.60	2.24	1.90	5.05	4.50	4.80	3.27	2.97	3.12
	Minimum	1.45	4.62	3.05	0.97	1.51	1.235	2.18	1.80	2.00	2.25	1.41	1.83
	Median	3.91	4.83	4.45	1.27	2.06	1.7	4.08	2.90	3.50	2.97	3.73	3.35
	SD	0.82	0.74	0.78	0.21	0.26	0.24	0.91	0.80	0.86	0.35	0.50	0.43
	*p*-value *	0.014						0.009					
	*p*-value **	0.014						0.020					
Na (mg/L)	Mean	132.63	216.73	176.42	27.84	47.90	37.87	158.67	107.1	132.90	83.57	73.65	78.65
	Maximum	192.27	329	260.65	35.85	54.90	45.4	252	159	205.50	102	86.80	94.4
	Minimum	27.74	192	109.85	24.66	35.20	29.95	78.40	52.80	65.60	67.50	38.50	53
	Median	142.87	201	172.45	25.59	49.80	37.4	162	101.50	131.75	83.20	76.25	79.85
	SD	41.17	36.16	38.67	3.88	7.37	5.63	61.32	29.60	45.46	10.46	14.82	12.6i
	*p*-value *	0.017						0.033					
	*p*-value **	0.031						0.014					
EC (mS/m)	Mean	44.20	150.87	97.54	9.13	43.88	26.51	92.92	94.44	93.65	74.07	66.84	70.45
	Maximum	54.10	229	141.55	14.20	49	31.6	143.10	119.70	131.40	84.80	72	78.4
	Minimum	28.60	135	81.8	5	38	21.5	52	60.70	56.35	55	54.10	54.55
	Median	47.10	143	95.05	9.50	44.50	27	90.50	94.40	92.45	81.60	68.25	74.95
	SD	7.55	23.67	15.61	3.02	4.02	3.52	32.22	13.83	22.92	12.75	5.81	9.28
	*p*-value *	0.015						0.063					
	*p*-value **	0.270						0.011					
Temperature (°C)	Mean	26.2	-		26.45	-		17.55	16.32	16.50	19.84	16.46	18.18
	Maximum	30	-		29.20	-		24.60	17.60	18.05	24.20	17.40	20.8
	Minimum	24.4	-		24.70	-		13.50	15.60	14.55	16.50	15.60	16.05
	Median	26.45	-		26.30	-		17.20	16.05	16.13	19.20	16.60	18
	SD	1.67	-		1.32	-		2.84	0.63	1.74	2.93	0.64	1.79
	*p*-value *	*-*			*-*			0.008					
	*p*-value **												

N = number of variables, * Cultivated, ** Uncultivated, *p*-value * (uncultivated vs. cultivated), *p*-value ** (seasonality, wet vs. dry period).

**Table 2:** Soil texture for cultivated and uncultivated wetland sites.

Texture	Mean (%) ± Std. Deviation		*p*-Value
	Cultivated	Uncultivated	
Sand	13.43 ± 15.32	73.63 ± 15.32	0.03
Silt	41.97 ± 10.42	17.02 ± 15.31	0.51
Clay	44.60 ± 14.58	9.35 ± 7.88	0.04

**Table 3:** Soil physico-chemical properties of cultivated and uncultivated sites.

Parameters	Statistics	March 2017		July 2017		March 2018		July 2018	
		Cult	Uncult	Cult	Uncult	Cult	Uncult	Cult	Uncult
EC (mS/m)	N	17	10	14	10	17	10	13	10
	Mean	57.4	31.7	14.2	11.2	25	19.8	27.4	16.5
	Maximum	84	85	19	22	44	19.8	84	20
	Minimum	11	12	9	2	16	3	2	14
	Median	62	23	14	11.5	22.5	19.8	17.3	17.5
	SD	21.1	23.9	2.6	6.8	8.2	11.1	29.8	2.3
	*p*-value	0.30		0.54		0.33		<0.01	
CEC (cmol(+)/kg)	N	17	10	14	10	17	10	13	10
	Mean	25.9	31.2	29.7	25.9	19.8	32.8	22.1	37.8
	Maximum	43.2	44.2	37.8	36.8	19.8	39.9	32.4	49.6
	Minimum	3.1	9.2	11.7	12.9	3	15.5	5.6	31.4
	Median	27.5	32.9	30.7	28.6	19.8	33.6	30.8	36.2
	SD	13.9	11.4	7.1	8.3	11.1	6.9	7.1	5.5
	*p*-value	0.98		0.65		0.83		0.69	
pH	N	-	-	-	-	17	10	13	10
	Mean	-	-	-	-	0.36	0.37	0.34	0.34
	Maximum	-	-	-	-	0.36	0.40	0.38	0.37
	Minimum	-	-	-	-	0.32	0.36	0.32	0.31
	Median	-	-	-	-	0.37	0.37	0.33	0.34
	SD					0.07	0.01	0.02	0.02
	*p*-value					0.13		0.59	
Organic matter (%)	N	-	-	-	-	17	10	13	10
	Mean	-	-	-	-	6.2	5.7	6.6	5.8
	Maximum	-	-	-	-	6.2	6	6.9	6.1
	Minimum	-	-	-	-	4.8	4.8	6.3	5.5
	Median	-	-	-	-	5.9	5.7	6.2	5.8
	SD	-	-	-	-	1.1	0.36	0.23	0.22
	*p*-value	-		-		0.08		0.52	
N-NO_3-_ (mg/kg)	N	17	10	14	10	17	10	13	10
	Mean	9.5	7.4	6.4	5.5	8.9	11.5	8.5	11.7
	Maximum	23.1	19.2	8	7.2	8.9	12.5	10.8	12.9
	Minimum	11.2	1.2	4.1	3.4	4.9	9.9	6.1	10.4
	Median	5.6	5.9	6.2	5.2	10.1	11.6	10.2	11.8
	SD	8.1	5.9	1.2	0.9	1.86	0.89	1.16	0.86
	*p*-value	0.05		0.08		0.01		0.02	
Total carbon (%)	N	17	10	14	10	17	10	13	10
	Mean	4.6	5.1	5.5	6.4	14.3	8.3	12.6	9
	Maximum	7.5	8.8	7.2	8	36.7	34.5	33.5	26.8
	Minimum	1	1.2	3.4	4.1	0.3	2.6	0.23	4.1
	Median	5.3	4.5	5.2	6.2	13.5	7.5	12.4	8.1
	SD	2.2	2.3	0.9	1.2	10.8	5.7	9.4	4.6
	*p*-value	0.94		0.22		0.03		0.92	
Total nitrogen (%)	N	17	10	14	10	17	10	13	10
	Mean	0.07	0.10	0.09	0.15	1.5	2.8	1.5	2.6
	Maximum	0.12	0.13	0.13	0.73	2.4	3.9	2.9	3.8
	Minimum	0.04	0.05	0.01	0.07	0.6	1.6	0.03	1.2
	Median	0.07	0.10	0.09	0.11	1.5	2.6	0.1	2.6
	SD	0.02	0.03	0.02	0.16	0.5	0.6	0.07	0.7
	*p*-value	0.76		0.03		0.17		0.06	

Cult = cultivated; Uncult = uncultivated; N = number of samples; - = no data.

## Data Availability

The data belongs to the University of the Witwatersrand, however the data can be available on request, but the intention of using the data must be for the purpose of conducting research, then the disclosure include the following: The title of the research or paper for which the specified data is to be used; The details of the institution and supervisory body or persons under the auspices of the research is undertaken; The assurance that no commercial gain will be received from the outcome from the research.

## Supplementary Materials

The following are available online at https://www.mdpi.com/1660-4601/18/2/561/s1, Table S1. Average monthly rainfall and maximum temperature for the 2017 and 2018 periods; Table S2. SAWQ guidelines accepted limits for agricultural use: irrigation; Table S3. Irrigation water classes for electrical conductivity and sodium adsorption ratio.
