# Supplementary material for: Assessing the Effects of Land Use on Surface Water Quality in the Lower uMfolozi Floodplain System, South Africa
Source: Int J Environ Res Public Health. 2021 Jan 11;18(2):561. doi: 10.3390/ijerph18020561 (PMC7827912; doi:10.3390/ijerph18020561)
Supplement: Supplementary file 1 [file ijerph-18-00561-s001.pdf]

Table 1. Average monthly rainfall and maximum temperature for the 2017 and 2018 periods.

Months	Monthly average rainfall (mm)		Monthly maximum temperature (°C)	
	2017	2018	2017	2018
JAN	126.6	29.4	30	31
FEB	179.2	140.4	30.3	29.3
MAR	46.6	66.4	30.6	30.2
APR	18.2	59.6	28.8	28.9
MAY	314	205.4	27.4	26.5
JUN	36.2	40	25.7	25.4
JUL	10.8	3	25.4	25
AUG	3.8	48.4	25.7	25.3
SEP	39.4	40.6	27.3	27.6
OCT	64.4	96	27.5	26.7
NOV	122	40.2	28.1	27.6
DEC	87.6	107.4	28.6	29.3

Source: https://www.weathersa.co.za/.

Table 2. SAWQ guidelines accepted limits for agricultural use: irrigation [1].

Parameters	Accepted Limits Guideline
**Ammonium (mg/l))**	5
Boron (mg/l)	0.5
Chloride (mg/l)	100
Fluoride (mg/l)	2
Nitrate (mg/l)	5
Nitrite (mg/l)	5
pH	8.4
SAR	2
Sodium (mg/l)	70
EC (mS/m)	40

Table 3. Irrigation water classes for electrical conductivity and sodium adsorption ratio [2].

Constituents	Fitness for Use			
	Good	Fair	Marginal	Unacceptable
EC (mS/m)	0–40	40–90	90–270	>270
SAR	0–1.5	1.5–3.0	3.0–5.0	>5.0

1. DWAF. South African Water Quality Guidelines. Volume 4: Agricultural Use. Department of Water Affairs and Forestry: 1996.
2. Koegelenberg, F. Irrigation User'; s Manual-Chapter 5: Water. *Agricultural Research Council, Silverton, South Africa* **2004**.
